# Toward New Modalities in VEP-Based BCI Applications Using Dynamical Stimuli: Introducing Quasi-Periodic and Chaotic VEP-Based BCI

**DOI:** 10.3389/fnins.2020.534619

**Published:** 2020-11-17

**Authors:** Zahra Shirzhiyan, Ahmadreza Keihani, Morteza Farahi, Elham Shamsi, Mina GolMohammadi, Amin Mahnam, Mohsen Reza Haidari, Amir Homayoun Jafari

**Affiliations:** ^1^Computational Neuroscience, Institute of Medical Technology, Brandenburg University of Technology Cottbus-Senftenberg, Cottbus, Germany; ^2^Department of Medical Physics & Biomedical Engineering, School of Medicine, Tehran University of Medical Sciences, Tehran, Iran; ^3^Research Center for Biomedical Technologies and Robotics (RCBTR), Tehran University of Medical Sciences, Tehran, Iran; ^4^Department of Biomedical Engineering, Faculty of Engineering, University of Isfahan, Isfahan, Iran; ^5^Section of Neuroscience, Department of Neurology, Faculty of Medicine, Baqiyatallah University of Medical Sciences, Tehran, Iran

**Keywords:** VEP-based BCI, chaotic stimuli, quasi-periodic stimuli, CCA, coherence

## Abstract

Visual evoked potentials (VEPs) to periodic stimuli are commonly used in brain computer interfaces for their favorable properties such as high target identification accuracy, less training time, and low surrounding target interference. Conventional periodic stimuli can lead to subjective visual fatigue due to continuous and high contrast stimulation. In this study, we compared quasi-periodic and chaotic complex stimuli to common periodic stimuli for use with VEP-based brain computer interfaces (BCIs). Canonical correlation analysis (CCA) and coherence methods were used to evaluate the performance of the three stimulus groups. Subjective fatigue caused by the presented stimuli was evaluated by the Visual Analogue Scale (VAS). Using CCA with the M2 template approach, target identification accuracy was highest for the chaotic stimuli (*M* = 86.8, *SE* = 1.8) compared to the quasi-periodic (*M* = 78.1, *SE* = 2.6, *p* = 0.008) and periodic (*M* = 64.3, *SE* = 1.9, *p* = 0.0001) stimulus groups. The evaluation of fatigue rates revealed that the chaotic stimuli caused less fatigue compared to the quasi-periodic (*p* = 0.001) and periodic (*p* = 0.0001) stimulus groups. In addition, the quasi-periodic stimuli led to lower fatigue rates compared to the periodic stimuli (*p* = 0.011). We conclude that the target identification results were better for the chaotic group compared to the other two stimulus groups with CCA. In addition, the chaotic stimuli led to a less subjective visual fatigue compared to the periodic and quasi-periodic stimuli and can be suitable for designing new comfortable VEP-based BCIs.

## Introduction

Electroencephalogram (EEG) is commonly used for EEG-based brain computer interfaces (BCIs) as a non-invasive and low-cost method for measuring the brain neural activities (Wolpaw et al., 2002; Lebedev and Nicolelis, 2006). BCI applications employing EEG use visual evoked potentials (VEPs) (Middendorf et al., 2000; Müller-Putz et al., 2005; Lee et al., 2006, 2008; Wang et al., 2006,Wang Y. et al., 2017; Martinez et al., 2007; Allison et al., 2008; Guo et al., 2008; Chen et al., 2015; Xie et al., 2017, 2018), which are responses of the visual system to visual stimuli. Various types of visual stimuli, such as flickering LED, can be decoded from the EEG activity of the visual cortex and used for diverse BCI applications (Takano et al., 2009; Lee et al., 2011; Gao et al., 2014; Kapgate and Kalbande, 2015).

In VEP-based BCIs, the target stimuli are identified by decoding all the gazed stimuli. The stimuli are required to be orthogonal or almost orthogonal in time-, frequency-, or code-based BCIs (Bin et al., 2009a). The oddball paradigm is an example of time-based BCIs, whereby the target stimuli are presented at different times and evoke event-related potentials (ERPs) like the P300 (Jin et al., 2005; Lee et al., 2008; McCane et al., 2015). Frequency-based BCIs may use visual stimuli that are modulated in time according to a sine wave with different temporal frequencies, which also evoke EEG responses at the same frequencies and their harmonics (Middendorf et al., 2000; Müller-Putz et al., 2005; Wang et al., 2006). Orthogonal visual stimuli in code-based BCIs are generated by random codes such as *m*-sequences, whereby different shifts of a modulating code have been used to evoke code-modulated VEPs (c-VEPs) (Nakanishi et al., 2014; Riechmann et al., 2016; Wei et al., 2016, 2018; Spüler, 2017; Liu et al., 2018; Shirzhiyan et al., 2019).

Due to their high decoding accuracy, external stimuli such as periodic flickers are commonly used in VEP-based BCIs evoking steady-state visual evoked potentials (SSVEPs) (Vialatte et al., 2010; Keihani et al., 2018). In SSVEP-based BCIs, the stimulus comprises a constant frequency that varies from low to high (1–100 Hz), which then leads to specific EEG responses that correlate with the stimulus frequency (Vialatte et al., 2010). Therefore, the gazed target stimuli could be identified from their EEG responses. However, among different frequency sets, the lower (1–12 Hz) and medium (13–16 Hz) ones lead to high subjective discomfort, fatigue, and possible epileptic seizures (Volosyak et al., 2011). Various dynamical approaches have also been used for improving SSVEP-based BCIs, such as dynamic stopping methods and the detection of SSVEP responses for higher information transfer rate (ITR) SSVEP-based BCIs (Yin et al., 2014; Jiang et al., 2018).

Visual stimuli have diverse dynamical patterns such as periodic, quasi-periodic, and chaotic. Biological systems also exhibit these dynamical behaviors (Attinger et al., 1966; Petrov et al., 1997; Suzuki et al., 2016) including non-oscillatory chaotic behavior, which is more complex than quasi-periodic oscillation (Camazine et al., 2003; Saha and Galic, 2018; Strogatz, 2018). Neuronal systems exhibit both complex oscillatory behavior (Llinás, 1988; Zhanabaev and Kozhagulov, 2013; Zhanabaev et al., 2016; Feng et al., 2017) as well as the non-oscillatory chaotic behavior that is seen in neurons (Aihara et al., 1984; Hong, 2011; Lv et al., 2016; Ma and Tang, 2017) and networks (Aihara, 1989; Freeman, 1992; Potapov and Ali, 2000; Rössert et al., 2015; Nobukawa and Nishimura, 2016) due to various underlying mechanisms (Hoebeek et al., 2010; Ishikawa et al., 2015). Stimuli with dynamical patterns such as chaotic behaviors are thus expected to be more in harmony with the visual system.

Natural visual stimuli rarely flicker at a constant rate, but rather exhibit more complex dynamics with quasi-periodic temporal characteristics (Kayser et al., 2003; Butts et al., 2007; Mazzoni et al., 2011). Natural visual stimuli are efficiently encoded by the visual system which is capable of processing and detecting information from complex natural environments (Blake and Lee, 2005; Mazzoni et al., 2011). These visual stimuli have similar spatial and temporal patterns resembling the 1/*f* amplitude spectrum, features that are encoded more efficiently by the retina and other components of the visual system (Atick and Redlich, 1992; Yoshimoto et al., 2017). In addition, chaotic patterns also follow the 1/*f* spectrum observed in natural scenes and phenomena (Relano et al., 2002; Molina et al., 2010). Quasi-periodic visual stimuli can generate phase-locked responses (Keitel et al., 2017; Obleser et al., 2017; Haegens and Golumbic, 2018) and also evoke responses with independent dynamics that correlate with their corresponding stimuli (Keitel et al., 2017). Temporal dynamics of the presented visual stimuli leads to adjustment of the visual system based on its inherent characteristics (Lasley and Cohn, 1981; Correa and Nobre, 2008). For these reasons, it is possible to assume that complex stimuli with dynamical temporal patterns such as quasi-periodic and chaotic may generate correlated responses which may lead to greater visual comfort for the viewer compared to the periodic stimuli.

One of the important issues in VEP-based BCI applications is the subjective visual fatigue caused by the flickering stimuli (Volosyak et al., 2011; Chang et al., 2014; Won et al., 2015). Periodic stimuli generating SSVEPs, due to their high contrast flashes, are not comfortable and can lead to subjective visual fatigue (Kardan et al., 2015; Xie et al., 2016). These periodic patterns may also lead to migraine headache (DeTommaso et al., 1999) or even epileptic seizures (Fisher et al., 2005). Studies have used various methods including the use of high-frequency stimuli rather than lower frequencies (Allison et al., 2010; Sakurada et al., 2015; Ajami et al., 2018), polychromatic stimuli (Chien et al., 2017), motion Newton’s rings and motion checkerboards (Xie et al., 2012, 2017; Yan et al., 2017; Han et al., 2018), and rhythmic pattern stimuli (Keihani et al., 2018) to minimize the subjective visual fatigue, which still remains an important problem in VEP-based BCI applications.

Utilization of visual stimuli with quasi-periodic and chaotic patterns that are closer to natural scenes in BCI applications requires further research. In our previous study, we used chaotic and pseudo-random *m*-sequence binary codes and found that chaotic codes lead to comparatively less fatigue (Shirzhiyan et al., 2019). In this study, we introduce a new kind of visual stimuli with quasi-periodic and chaotic characteristics to evoke distinct visual potentials in normal subjects for their possible application in VEP-based BCIs. For comparison, we used periodic stimuli commonly employed in SSVEP-based BCI applications and also compared subjective visual fatigue caused by these three groups of stimuli.

## Materials and Methods

**FIGURE 1:**
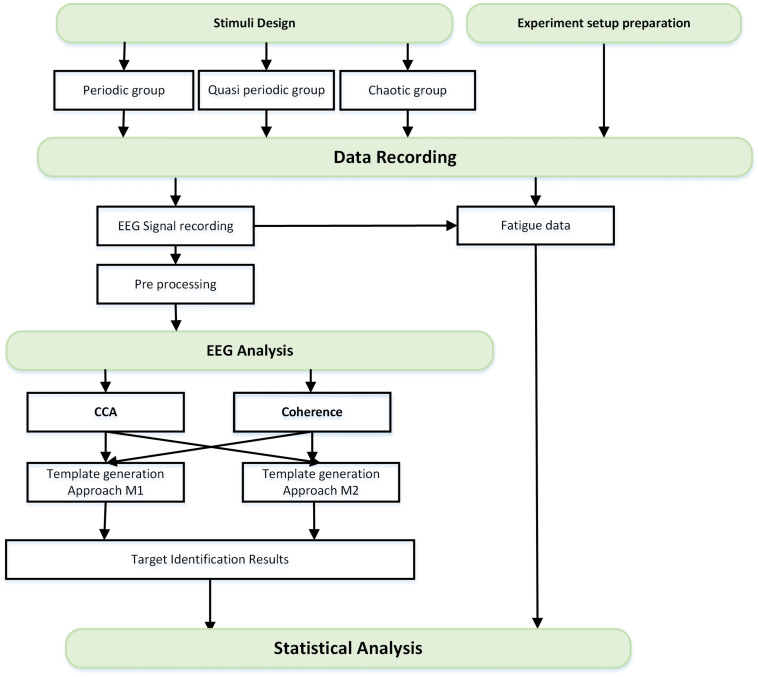
Flowchart of the study roadmap.

In this study, first of all, the stimulus groups were designed and proper setup for the experiment was prepared. The data recording step started with EEG and behavioral data (fatigue data) recording from normal subjects. After preprocessing of the EEG data, the data analysis was done to decode the presented stimuli from their corresponding data using canonical correlation analysis (CCA) and coherence analysis methods. These two methods calculate the similarity of templates with EEG signals where the stimuli could be considered as templates (template generation approach M1) or obtained from a training dataset (template generation approach M2). Finally, the target identification results using the above-mentioned methods and the fatigue data were analyzed separately. The flowchart of this study is presented in Figure 1.

### Study Participants

Our study was announced in the faculties of medicine and biomedical engineering *via* notice boards as well as in students’ social media groups. Forty-eight volunteers were initially enrolled based on the inclusion criteria (normal or corrected vision with no history of head trauma and without current use of drugs). All subjects were informed about the study aims and procedure of signal recording and were allowed to leave the experiment at any point if they wished. Thirty-eight subjects participated in all the sessions (18 females), aged 20–33 years old (23.01 ± 4.32). The remaining 10 subjects did not participate in all the sessions or left the session due to urgent work, and so their data were excluded from the study. Written informed consent was signed by the participants before joining the study in accordance with the Declaration of Helsinki. The study was approved by the Office of Research Review Board and the Research Ethics Committee of the Tehran University of Medical Sciences, with LREC protocol number IR.TUMS.REC.1394.2110.

### Stimuli

We used visual stimuli consisting of modulating the brightness of a red color LED measuring 4 cm × 4 cm according to three different temporal patterns: periodic, quasi-periodic, or chaotic. Each of these three categories had four different target stimuli that had their orthogonal characteristics. All the stimuli were generated using MATLAB software (Release 2016b, The MathWorks, 193 Inc., Natick, MA, United States).

#### Periodic Stimuli

**FIGURE 2:**
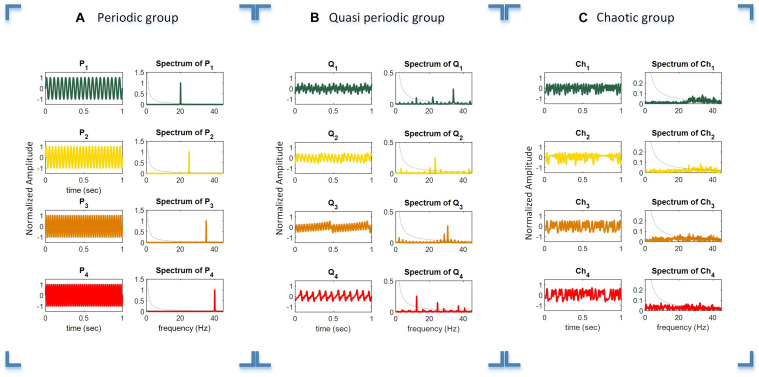
Time duration, amplitude, and frequency spectrum of the periodic **(A)**, quasi-periodic **(B)**, and chaotic **(C)** stimulus groups. *Columns 1* and *2* in each plot show the relevant waveforms and spectra of the stimuli of each group. For better illustration of the waveforms, they are shown in a 1 s timescale, while the total duration of stimuli was 6 s.

For generating the periodic stimuli, we used four sine waves at the target frequencies of *f*_1_−*f*_4_ (20, 25, 35, and 40 Hz), as shown in Equation 1 and schematically illustrated in Figure 2A. It can be seen that the simple periodic stimulus group (P_1_ – P_4_) had constant frequencies and that their spectrum was sparse in the frequency domain representation.

(1) $$\begin{array}{c} P_{i}=\sin\left(2\pi f_{i}t\right).t=0:6\text{sec}.f=[20\text{Hz}. 25\text{Hz}.\\ 35\text{Hz}. 40\text{Hz}] \end{array}$$

#### Quasi-Periodic Stimuli

A sine-circle map was used to generate four quasi-periodic stimuli (Essl, 2006). Equation 2 models the sinusoidal oscillators that were perturbed by non-linear function.

(2) $$\theta_{n+1}=\theta_{n}+\Omega -K/2\pi \sin\left(2\pi \theta_{n}\right)$$

where Ω is the frequency ratio and *K* is the coupling length of non-linear perturbation.

If the frequency ratio Ω is a rational number (*p*/*q*) with *p* and *q* ∈ *N* (natural numbers), the map shows periodic behavior. For irrational numbers of Ω and appropriate parameters of *K*, the behavior of the sine-circle map is called quasi-periodic oscillation (Essl, 2006).

Quasi-periodic stimuli were generated using a sine-circle map by considering the parameter *K* = 0.5 and then Ω were selected as irrational numbers $\left(\sqrt{5}-1\right)/2$, $\sqrt{3}-1$, $\sqrt{3}/2,$ and $\sqrt{2}/9$, where the sine-circle map showed quasi-periodic behaviors. These parameters were used to generate quasi-periodic stimuli Q_1_, Q_2_, Q_3_, and Q_4_. After that, the generated sequences from the sine-circle maps were considered as a time series with a sampling frequency of 90 Hz. Thus, each sample of the sequences was applied for a duration of 1/90 ms. The waveform and spectrum of the quasi-periodic stimuli are shown in Figure 2B. The waveform and spectrum of the quasi-periodic stimuli were more complex compared to the periodic stimulus group.

#### Chaotic Stimulus Group

For generating chaotic stimuli, we used a logistic map which is a one-dimensional map capable of generating chaotic signals with low cross-interferences. This map is seen in most of the natural phenomena and population growth of biological species (Costantino et al., 1997), as defined in Equation 3.

(3) x(i+1)=Ax(i)(1-x(i))

where *x* is in the interval of [0 1] and indicates the ratio of an existing population to the maximum possible population, *x*(0) as the initial value of *x*, and *A* is the rate of reproduction and starvation that is in the interval of [0 4]. This simple map could generate chaotic dynamics in some values of parameter *A* generally between 3.5 and 4 (May, 1976). Parameter *A* was chosen in a way that the logistic map exhibited chaotic behaviors for generating four chaotic sequences and were then presented at the rate of 90 Hz as *A* = 3.982, 3.885, 3.987, and 4, respectively. In this way, four different chaotic stimuli, Ch_1_, Ch_2_, Ch_3_, and Ch_4_, were generated by the logistic map.

Figure 2C shows the waveforms of four chaotic stimuli with their amplitude spectra.

The dotted black curved line in the amplitude spectra plots shows the 1/*f* line, where *f* is the frequency vector (horizontal axis). It can be seen that the amplitude spectra of the stimuli in the chaotic group are closer to the 1/*f* spectrum line compared to the quasi-periodic and periodic stimulus groups. Please note that for better illustration, only 1 s of the total 6 s duration of every stimulus is shown in the plot.

### Auto- and Cross-Correlation Function of Target Stimuli

**FIGURE 3:**
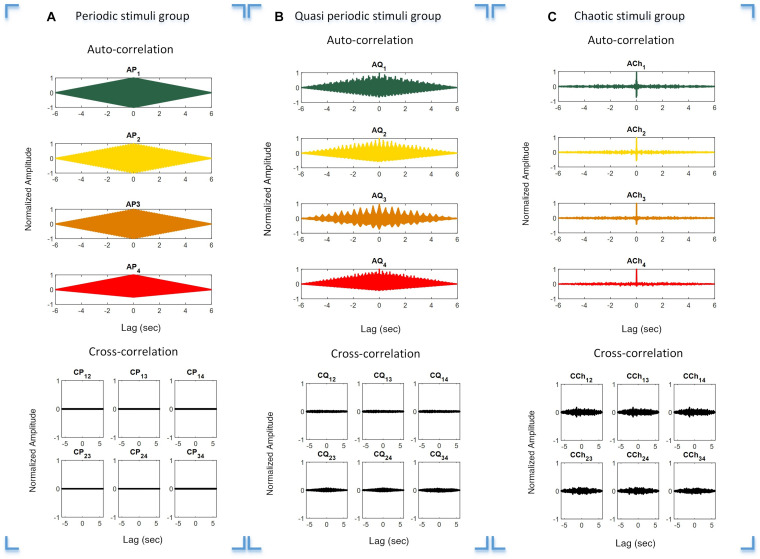
Auto- and cross-correlation functions of the three stimulus groups. Plot **(A–C)** Correlation functions over different time lags of the periodic **(A)**, quasi-periodic **(B)**, and chaotic **(C)** groups. *Rows 1* and *2* in each plot show the auto- and cross-correlations of the target stimuli in each category, respectively.

The auto- and cross-correlation functions of the periodic, quasi-periodic, and chaotic stimulus groups were calculated to investigate their individual orthogonal characteristics. This was done to verify overlapping characteristics in order to avoid interference between the target stimuli. The auto- and cross-correlation functions of the periodic, quasi-periodic, and chaotic groups are presented in Figures 3A–C, respectively. It is obvious that the auto-correlation function of each target stimulus group is high, while within-group cross-correlations of stimuli with others is comparatively low.

### Stimulus Presentation Paradigm

**FIGURE 4:**
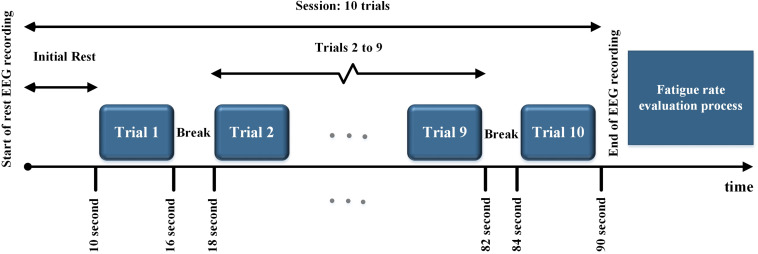
Stimulus presentation paradigm. All the stimuli in each group were presented in 10 trials. After each trial, a 2 s rest time was considered. At the end of each session, the fatigue rate was evaluated.

All the subjects were presented with periodic, quasi-periodic, and chaotic stimuli in a single lab visit. They were presented with each of the periodic (P_1_ – P_4_), quasi-periodic (Q_1_ – Q_4_), and chaotic (Ch_1_–Ch_4_) stimuli as 12 different sessions. The total duration of each session (for each stimulus) was 90 s consisting of 10 trials. In each trial (6 s duration), the same stimulus was presented to the subject with a 2 s rest time in between the trials. An initial rest of 10 s was included in each session. After each session (90 s), the subjective fatigue was evaluated (see below). The maximum duration of a whole stimulus presentation paradigm including rest time was approximately 30 min. The stimulus presentation paradigm is shown in Figure 4.

The subjects were informed before the experiment that they will be asked to evaluate their own visual fatigue by considering the amount of tiredness and discomfort caused by gazing at the stimuli. They were asked to grade their visual fatigue level by choosing a number between 0 for no fatigue and 10 for the highest fatigue level. After each session (90 s), the subjects were asked to self-report the level of visual fatigue caused by stimulation. Their fatigue rate was recorded and they were asked for permission to start the next session. The order of presentation of stimuli in all groups was randomly distributed for all subjects to avoid possible bias in subjective visual fatigue caused by the order of presentation.

### Fatigue Evaluation Process

The level of subjective visual fatigue was measured using graded values of the Visual Analogue Scale (VAS), which is suitable for grading continuous phenomena (Aitken, 1969). The VAS is a subjective estimation method for quantifying a feeling and attitude which is hard to estimate directly (Gift, 1989; Grant et al., 1999; Crichton, 2001; Tseng et al., 2010; Klimek et al., 2017). This scale is mostly used in clinical research for measuring the intensity of various symptoms (Paul-Dauphin et al., 1999) such as pain (Bijur et al., 2001). It is commonly used in BCI applications for the evaluation of a patient’s motivation and mood (Holz et al., 2013), the level of subjective fatigue (Guger et al., 2013; Käthner et al., 2014), pain (Choi, 2017), discomfort (Verwulgen et al., 2018), and control ability (Chumerin et al., 2012).

### Signal Recording Setup

The EEG signals were recorded using g.USB Amp with a sampling rate of 1,200 Hz. Four active g.Ladybird electrodes were placed at Oz, O1, O2, and Pz positions on the scalp of the subjects where the visual evoked potentials have maximum amplitude (Bin et al., 2011; Aminaka et al., 2015). Fpz and right earlobe were used as the ground and reference electrodes, respectively. An online bandpass filter with cutoff frequencies of 0.05 and 120 Hz was applied.

The generated stimuli were applied to a custom-made digital-to-analog converter (DAC) board as a stimulus presenter box (shown in Supplementary Figure 1) for driving an LED. The LED was placed at a distance of 70 cm from the subject. The trigger output of g.USB Amp (start time of EEG recording) and the output of a Texas Instruments optical sensor (visual stimuli) were sent to National Instruments (NI) DAQ. Details of the signal recording setup are reported in our previous studies (Keihani et al., 2018; Shirzhiyan et al., 2019).

### Data Processing

The signal analysis procedure was carried out for the recorded responses for each stimulus group (periodic, quasi-periodic, and chaotic) (Supplementary Figure 2) separately to compare the results of the three different groups. Tenfold cross-validation was used as our validation method. In this method, nine-tenths of the trials were used as the training data and one-tenth was used as the testing data. The training data was used for template generation and the testing data was used for target identification.

#### Preprocessing

The recorded trigger from g.USB Amp and sensor output and the presented stimuli in NI DAQ were used for the detection of the beginning of each trial and then each trial EEG data was extracted. A zero-phase eighth-order band pass filter with cutoff frequencies of 1 and 50 Hz was applied for all the trials.

#### Processing (CCA and Coherence Analysis)

To analyze the EEG data, we used two methods that are commonly employed in BCI studies: CCA and coherence analysis (Zhang et al., 2013, 2014; Vaid et al., 2015). These methods measure the amount of correlation in the time and frequency domains, respectively.

Canonical correlation analysis is a multivariable data analysis method that measures the underlying time domain correlation between two multidimensional signals and attempts to reveal a linear time domain correlation by maximizing the correlation of the two signals (Lin et al., 2006). CCA has been successfully used in target identification and in the analysis of visual evoked potentials (Lin et al., 2006; Bin et al., 2009b, 2011). Equation 4 defines the CCA coefficient of variables *x* and *y*, where *E*(*x*^*t*^*y*) stand for the covariance of *x* and *y* and *E*(*x*^*t*^*x*) and *E*(*y*^*t*^*y*) represent the variance of *x* and *y*, respectively. This method finds the canonical correlation vectors *W*_*x*_ and *W*_*y*_ for two multidimensional variables, *x* and *y*, by maximizing their canonical covariants.

(4) $$\underset{w_{x}.w_{y}}{\text{Max}\rho \left(x.y\right)=}\frac{E\left(x^{t}y\right)}{\sqrt{E\left(x^{t}x\right)E\left(y^{t}y\right)}}$$

Coherence analysis has been used to investigate the synchronization process of brain regions (Nunez et al., 1997; Lachaux et al., 1999; La Rocca et al., 2014). In addition, it has been used as a feature extraction and target identification method in BCI applications (Gysels and Celka, 2004; Krusienski et al., 2012; Liew et al., 2015). The coherence of two signals is sometimes called magnitude-squared coherence, as shown by Equation 5 which defines the amount of coherence of two signals at a specific frequency.

(5) $$C_{xy}\left(f\right)=\frac{{\left|S_{xy}\left(f\right)\right|}^{2}}{S_{xx}\left(f\right)S_{yy}\left(f\right)}$$

where *S*_*xx*_ and *S*_*yy*_ are the power spectral densities of variables *x* and *y*, respectively, and *S*_*xy*_(*f*) is the cross power spectral density between *x* and *y* (Sanei and Chambers, 2007).

In this study, variable *x* is the template and *y* is the EEG signal. In BCI studies, it is common to use either the presented stimuli or the average EEG signal of the training session trials to generate the template. Therefore, in this study, we used two approaches for generating templates. In the first approach (M1), the stimuli were used as templates, while in the second approach (M2), we attempted to extract templates from the training dataset.

#### Template Generation

Templates were generated by using the two aforementioned approaches as described below.

##### Approach 1 (M1): using the presented stimuli as templates

The target stimuli were resampled to the sampling frequency of the EEG responses (1,200 Hz) and zero-padded the resampled target stimulus *i* by lag time *D*_*i*_. The lag time *D*_*i*_ represents the systematic lag for the presented stimuli and was calculated by cross-correlating the target stimuli with the grand averaged EEG responses and determining the time lag that yielded the maximum cross-correlation values.

This step was not important for coherence analysis because the magnitude-squared coherence was not sensitive to the time lag between templates (stimuli) and responses, while the CCA coefficients were maximum where the lag was considered.

##### Approach 2 (M2): generating templates using the training dataset

In VEP-based BCI studies, it is also common to create templates using the EEG signals from a subset of the data (i.e., a training dataset) instead of the stimulus waveform itself, as this approach allows capturing information related to the non-linear processing of the system. Given that we had access to the training dataset, we used this approach to generate templates by EEG data from the training dataset.

This approach included extracting the EEG responses from *r* trials in the training set, $X_{{\text{Train}}_{i}}^{r\times m\times n}$, and averaging over *r* trials to generate the template for stimulus *i*, $T_{i}^{m\times n}$, where *m* is the number of channels and *n* is the number of samples per trial.

#### Target Identification

After generating templates separately for the targets in each group, the CCA coefficient and coherence were calculated by Equations 4 and 5, where *T* was considered as variable *x* and the EEG response was considered as variable *y*. For template generation using the M1 approach, all the trials were separately considered as testing trials, while 10-fold cross-validation was considered for template generation using the M2 approach. Here, ninefold of the dataset was considered as the training dataset and the one remaining fold was considered as the testing trial. Therefore, all the trials were tested once. The details of both the approaches of target identification are given below.

For the CCA method:

(1) Extraction of testing trials $X_{\text{Test}}^{m\times n}$, where *m* and *n* are the channel numbers and samples in a trial, respectively.
(2) Calculation of the CCA coefficient of templates *T*_*i*_ and $X_{\text{Test}}^{m\times n}$ as vector P_*i*_^1×*m*^.
(3) Calculation of the mean value of ${\text{P}}_{i}^{1\times m}$ to create the feature vector.
(4) Selection of the maximum value of feature vector.

For the coherence method:

(1) Extraction of testing trials $X_{\text{Test}}^{m\times n}$, where *m* and *n* are the channel numbers and samples in a trial, respectively.
(2) Calculation of the coherence function of templates *T*_*i*_ and $X_{\text{Test}}^{m\times n}$ for obtaining the vector *C*_*i*_(*f*).
(3) Extraction of the coherence coefficient from vector of *C*_*i*_(*f*) in the target frequencies or a specific frequency band.
(a) Periodic group: the target frequencies in the periodic group were the target frequencies of the presented stimuli as shown in Figure 2A.
(b) Quasi-periodic group: the dominant frequencies of the presented stimuli are shown in the spectra of stimuli in Figure 2B.
(c) Chaotic group: the total frequency band of the chaotic stimuli as shown in Figure 2C.
(4) Calculation of the mean value of $C_{i}^{m\times f}$ to create the feature vector.
(5) Selection of the maximum value of the feature vector.

**FIGURE 5:**
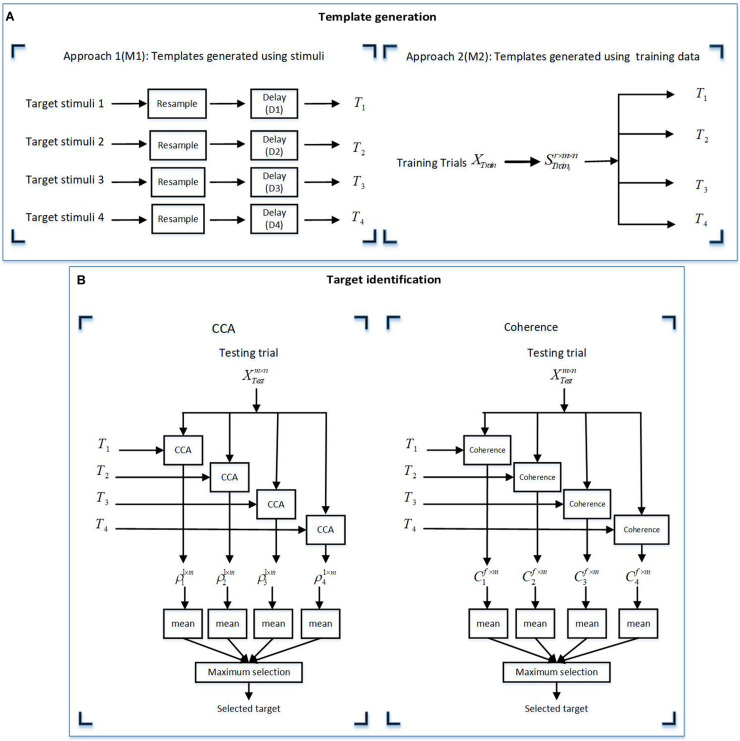
Illustration of the two methods for **(A)** template generation using approaches M1 and M2. **(B)** Target identification using the canonical correlation analysis (CCA) and coherence methods. **(A)** The templates for the CCA and coherence methods were derived by two approaches: M1 (using target stimuli) and M2 (using training data). **(B)** For the target identification, the derived templates from the template generation approaches **(A)** were used for analysis by the CCA and coherence methods. In this figure *m*, *n*, and *r a*re the channel numbers, samples in a trial, and the trial numbers in a training dataset, respectively. *f* represents the frequency vector in a specific frequency band in each stimulus group. *X*_*Train*_ are all training trials and *S*_*train_i*_ represents trial response to the *i*th stimulus in each stimulus group.

Figure 5 schematically shows the template generation and target identification processes.

### Statistical Analysis

Statistical analysis was done with SPSS software (version 16.0, SPSS Inc., IBM Corp., Chicago, released 2011) for comparing the analysis methods and also for evaluating the subjective visual fatigue rate between the three groups of stimuli (periodic, quasi-periodic, and chaotic).

#### Statistical Analysis of Accuracies

Three-way repeated measures ANOVA was used to test the effects of three factors—methods (CCA and coherence), approaches (M1 and M2), and stimulus groups (periodic, quasi-periodic, and chaotic)—with assumed sphericity (significance level α = 0.05). Confidence intervals were adjusted by Bonferroni correction for pairwise comparisons.

#### Statistical Analysis of Fatigue Rates

##### Within-group analysis of fatigue rates

To compare the VAS scores across stimuli within each stimulus type (periodic, quasi-periodic, and chaotic), the Friedman test (significance level α = 0.05) was used. Then, the scores for each pair of stimuli were compared using the Wilcoxon signed-rank test, with a Bonferroni-corrected alpha set to 0.008.

##### Between-group analysis of subjective visual fatigue rates

For comparison of the subjective visual fatigue caused by the periodic, quasi-periodic, and chaotic groups, the VAS scores of each group were averaged for the four sessions for each stimulus type for each subject and were then compared using the Friedman test (significance level α = 0.05). The Wilcoxon signed-rank test with Bonferroni correction was used for the comparison of each pair while α was set at 0.0168.

## Results

### Accuracy Analysis Results

**FIGURE 6:**
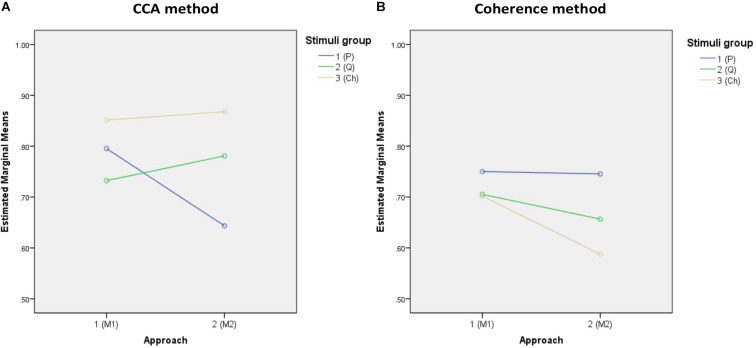
Marginal estimated means of all accuracies. **(A,B)** Marginal means of the accuracies of both approaches for the CCA and coherence methods, respectively. **(C,D)** Marginal means of the target identification accuracies of the stimulus groups for approaches M1 and M2, respectively.

The descriptive statistics of all accuracies of the three stimulus groups (periodic, quasi-periodic, and chaotic) obtained from two different methods (CCA and coherence) and template generation approaches (M1 and M2) are reported in Table 1, and Figure 6 shows the estimated marginal means plots of accuracies.

**TABLE 1:** Descriptive statistics of all accuracies obtained by two methods (CCA and coherence analysis) with two template generation approaches (M1 and M2) in three different stimulus groups.

Method	Approach	Stimulus group	Mean (%)	Standard error (%)	95% Confidence interval	
					Lower bound (%)	Upper bound (%)
CCA	M1	P	79.5	2.4	74.8	84.3
		Q	73.2	2.3	68.5	77.9
		Ch	85.1	2.2	80.6	89.7
	M2	P	64.3	1.9	60.5	68.2
		Q	78.1	2.6	72.8	83.4
		Ch	86.8	1.8	83.1	90.4
Coherence	M1	P	75.0	2.2	70.5	79.5
		Q	70.5	2.7	65.1	76.0
		Ch	70.2	2.7	64.7	75.7
	M2	P	74.5	2.5	69.5	79.6
		Q	65.7	2.7	60.1	71.2
		Ch	58.8	3.0	52.7	64.8

*CCA, canonical correlation analysis; M1, using target stimuli; M2, using training data; P, periodic; Q, quasi-periodic; Ch, chaotic.*

The 2 (analysis method) × 2 (template approach) × 3 (stimulus group) ANOVA on target identification accuracy revealed significant main effects of analysis method [*F*(1,37) = 60.253, *p* = 0.0001, $\eta_{\text{p}}^{2}$ = 0.620] and template approach [*F*(1,37) = 28.56, *p* = 0.0001, $\eta_{\text{p}}^{2}$ = 0.435], but not stimulus group [*F*(1,37) = 1.143, *p* = 0.324, $\eta_{\text{p}}^{2}$ = 0.030], with overall higher accuracy for the CCA method relative to coherence and higher accuracy for the M1 template approach relative to M2.

However, these effects were qualified by significant two-way interactions between analysis method and stimulus group [*F*(2,74) = 47.0009, *p* = 0.0001, $\eta_{\text{p}}^{2}$ = 0.56] and template approach and stimulus group [*F*(2,74) = 8.776, *p* = 0.0001, $\eta_{\text{p}}^{2}$ = 0.192]. The interaction between analysis method and template approach was not significant [*F*(1,37) = 3.695, *p* = 0.062, $\eta_{\text{p}}^{2}$ = 0.091]. Finally, the three-way interaction was significant [*F*(2,74) = 35.74, *p* = 0.0001, $\eta_{\text{p}}^{2}$ = 0.491]. To decompose the three-way interaction, we examined the effects of stimulus group for each analysis method and template separately and conducted pairwise comparisons between the stimuli groups.

For CCA and template approach M1, accuracy was higher for the chaotic stimuli (*M* = 85.1, *SE* = 2.2) than for periodic (*M* = 79.5, *SE* = 2.4, *p* = 0.0001) and quasi-periodic (*M* = 73.2, *SE* = 2.3, *p* = 0.008), with quasi-periodic also being lower than periodic (*p* = 0.0001). With template approach M2, CCA accuracy was again higher for the chaotic group (*M* = 86.8, *SE* = 1.8) compared to the other two groups, the accuracy now being better for the quasi-periodic (*M* = 78.1, *SE* = 2.6) than for the periodic stimuli (*p* = 0.0001) (Figure 6A).

For coherence analysis, using M1 template approach, multiple comparison with α = 0.0168 did not show significant differences between the periodic (*M* = 75.0, *SE* = 2.2), chaotic (*M* = 70.2, *SE* = 2.7), and quasi-periodic (*M* = 70.5, *SE* = 2, *p* > 0.0168) stimulus groups. Using M2 template approach, the periodic group (*M* = 74.5, *SE* = 2.5) showed higher accuracy than did the chaotic group (*M* = 58.8, *SE* = 3, *p* = 0.001), and the quasi-periodic group (*M* = 65.5, *SE* = 2.7) did not significantly differ from the other groups (*p* > 0.02) (Figure 6B).

### VAS Scores Analysis Results

**FIGURE 7:**
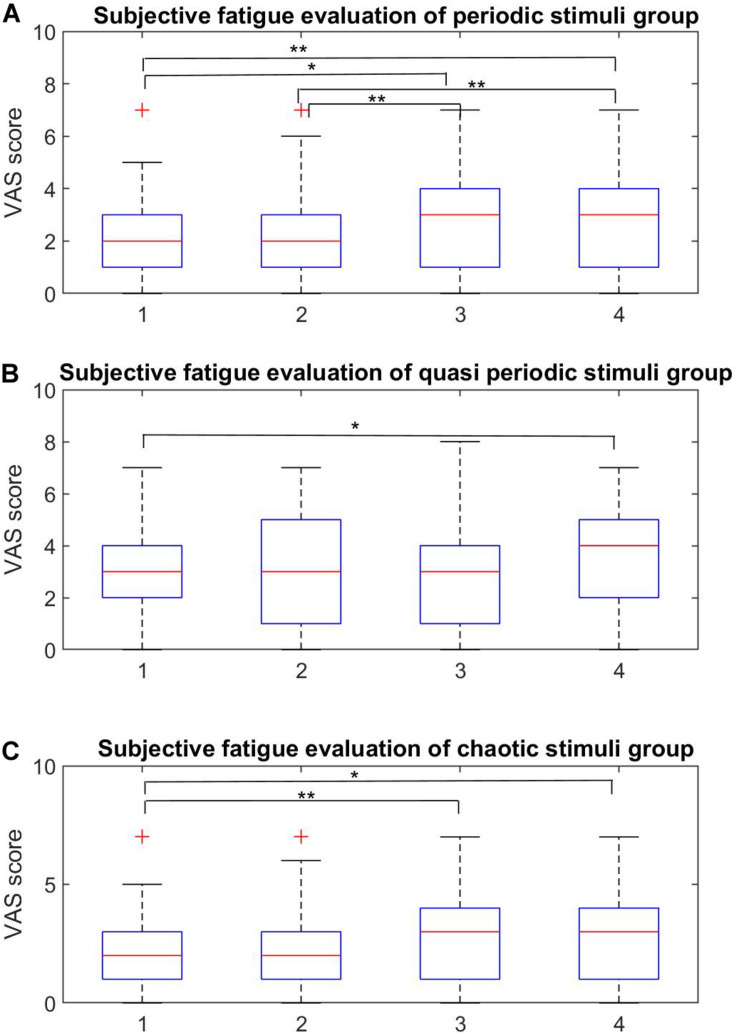
Within-group subjective fatigue evaluation. **(A–C)** Within-group analysis of the fatigue rates for the periodic, quasi-periodic, and chaotic groups, respectively. **(A)** In the periodic group, the pairs of (P_1_, P_3_), (P_1_, P_4_), (P_2_, P_4_), and (P_3_, P_4_) showed significant differences. Higher frequencies led to lower fatigue rates. **(B)** There was only one significant difference within Q_1_ and Q_4_ in the quasi-periodic group. **(C)** The chaotic stimulus group showed significant difference between the Ch_1_–Ch_3_ and Ch_1_–Ch_4_ pairs (*p < 0.001, **p < 0.0001).

**FIGURE 8:**
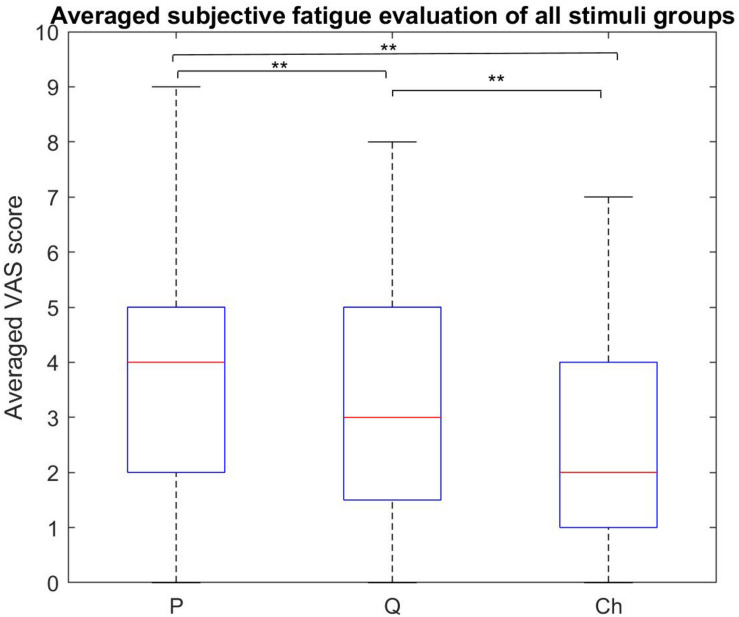
Subjective fatigue rates comparison between all the stimulus groups. The periodic group had the highest fatigue rate compared to the other two groups. The chaotic group had the least fatigue rate compared to the periodic and quasi-periodic groups (**p < 0.0001, *p < 0.016).

The subjective fatigue VAS scores for the four stimuli within each stimulus group are shown in Figure 7 and the averaged scores for each stimulus group are shown in Figure 8.

#### Results of Within-Group Analysis of the Periodic Group

The Friedman test showed significant differences in the VAS scores across stimuli in the periodic group [χ^2^(3) = 37.857, *p* = 0.0001]. Participants reported on average highest subjective fatigue scores for P_1_ (20 Hz) (*M* = 4.92, *SE* = 0.38) and P_2_ (25 Hz) (*M* = 4.5, *SE* = 0.30), which did not differ from each other (*Z* = 1.99, *p* = 0.046). The scores for P_3_ (30 Hz) were significantly lower (*M* = 3.2, *SE* = 0.22) than for P_2_ (*p* < 0.001), and P_4_ had the lowest scores (*M* = 2.71, *SE* = 0.28).

#### Results of Within-Group Analysis of the Quasi-Periodic Group

The Friedman test showed significant differences among the VAS scores of stimuli in the quasi-periodic group [χ^2^(3) = 14.848, *p* = 0.002]. Q4 (*M* = 3.65, *SE* = 0.32) caused relatively higher fatigue scores compared to Q1 (*M* = 3.08, *SE* = 0.30, *Z* = 2.85, *p* = 0.004) in the pairwise comparison of within-group quasi-periodic stimuli. Q2 (*M* = 3.13, *SE* = 0.30) and Q3 (*M* = 3.05, *SE* = 0.326) did not differ significantly from the others.

#### Results of Within-Group Analysis of the Chaotic Group

The Friedman test showed significant differences in the VAS scores for the stimuli in the chaotic group [χ^2^(3) = 20.125, *p* = 0.0001]. Ch_1_ (*M* = 2.02, *SE* = 0.28) had lower fatigue scores compared to Ch_3_ (*M* = 2.78, *SE* = 0.31, *Z* = 3.53, *p* = 0.0001), and Ch_4_ (*M* = 2.84, *SE* = 0.30, *Z* = 2.79, *p* = 0.005). Ch_2_ (*M* = 1.78, *SE* = 0.28) did not differ from Ch_1_ and Ch_4_.

Details of the *p*-values for subjective fatigue comparisons using the Friedman test with Bonferroni correction are presented in Supplementary Table 1.

#### Between-Group Analysis of Subjective Visual Fatigue Rate

The Friedman test showed that the subjective visual fatigue scores differed across the three stimulus groups [χ^2^(2) = 28.69, *p* = 0.0001]. Participants reported higher subjective fatigue for the periodic stimuli compared to the quasi-periodic (*Z* = 2.931, *p* = 0.003) and chaotic stimuli (*Z* = 4.429, *p* = 0.0001). In addition, they also felt higher fatigue for the quasi-periodic stimuli compared to the chaotic group (*Z* = 3.466, *p* = 0.001).

## Discussion

In this study, for the first time, we used quasi-periodic and chaotic stimuli with different orthogonal characteristics and compared them with periodic stimuli commonly employed in SSVEP-based BCIs that use EEG data. We also compared the level of subjective visual fatigue caused by these three stimuli on young adult participants. For this purpose, three groups of visual stimuli with different temporal dynamics (periodic, quasi-periodic, and chaotic) from simple sinusoidal frequencies to complex stimuli were generated from sine-circle and logistic maps and used for evoking visual potentials.

Periodic stimuli have been used for years in VEP generation for eliciting SSVEP responses that are known for their relatively high ITR, less training time (Parini et al., 2009), and practical BCI applications (Lalor et al., 2005; Muller-Putz and Pfurtscheller, 2008; Bin et al., 2009b; Yin et al., 2015; Lin et al., 2016; Wang Y.T. et al., 2017; Wang et al., 2018). Our results showed that the introduced dynamical visual stimuli (quasi-periodic and chaotic stimulus groups) could also evoke discriminative responses and can have even better target identification accuracies than the periodic visual stimulus group using the CCA method. In addition, compared to the other stimulus groups (periodic and quasi-periodic), the obtained accuracy values of target identification for the chaotic group by employing the CCA method for template generation approaches M1 (stimuli waveforms considered as a template) and M2 (templates generated from training EEG dataset) were the highest, with values of *M* = 86.78%, *SE* = 1.8% and *M* = 85.1%, *SE* = 2.2%, respectively. The results of the M1 approach for the periodic, quasi-periodic, and chaotic stimuli indicate that their corresponding EEG responses correlated with their stimuli waveforms. It has been reported that the temporal structures of quasi-rhythmic stimuli are reflected in the brain responses in the visual cortex (Keitel et al., 2017).

### Auto- and Cross-Correlation Function of Stimulus Groups

The stimuli in the chaotic and quasi-periodic groups as well as in the periodic group had orthogonal characteristics (Figure 3). This was demonstrated by the fact that the pairwise cross-correlation values between the stimuli were less than the auto-correlation values for the target stimuli (Figure 3). The cross-correlation functions showed that the stimuli within each stimulus group were not correlated because their cross-correlation values were close to zero. This meant that these stimuli were nearly orthogonal and the interference between the stimuli would be reduced in possible BCI applications. It is worth noting that the chaotic group’s auto-correlation exhibited a Dirac-like function, meaning that while these stimuli were orthogonal, they did not correlate with themselves. This feature was absent in the periodic and quasi-periodic groups, which required a higher level of synchronization between the visual stimuli and their EEG responses. The concept of Dirac-like auto-correlation function has been used in code-modulated BCIs for generating uncorrelated target stimuli from one code by the process of shifting (Bin et al., 2011).

### Cross-Correlation Function of Stimuli and Responses

**FIGURE 9:**
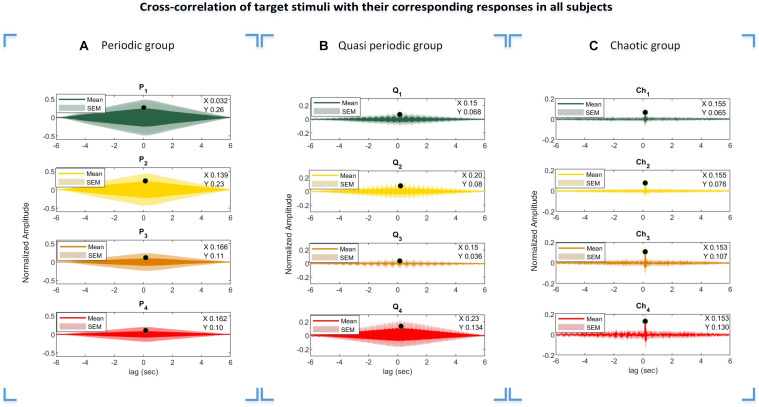
Cross-correlations of the periodic, quasi-periodic, and chaotic stimuli with their corresponding EEG responses. Results of the periodic **(A)**, quasi-periodic **(B)**, and chaotic **(C)** groups in all subjects. The lag of maximum correlation values in the plots were considered as delay time (*D*_*i*_, *i* = [1 2 … 4]) in template generation using the M1 approach. *X-* and *Y*-values represent the lag *black dot* values where the cross-correlation function of the stimuli and their corresponding responses are maximum, respectively.

The cross-correlation function of the presented stimuli and their corresponding responses suggest that the visual pathway system serves as an input and the evoked potentials as the system output. The lag of maximum of the cross-correlation function is considered as a system delay, which was used in our analysis especially in the CCA method for the M1 approach to generate templates from the presented stimuli. Due to the periodic and semi-periodic nature of the cross-correlation function (as seen in Figures 9A,B), compensating for the delay in templates was not necessary. However, compensating for the delay was vital for the chaotic group because the chaotic stimuli correlated with their corresponding responses in a specific time delay (Figure 9C). For the CCA method using template generation from the training dataset (M2 approach), the time delay compensation was not needed as the inherent time delay was embedded in the templates extracted from the training dataset.

### Target Identification Results Comparison

The highest accuracy for the chaotic group was obtained by CCA using the M2 approach, while the lowest accuracy was obtained by the coherence method using the M2 approach. The much lower accuracy seen with the chaotic stimuli using coherence analysis may be explained by the fact that the spectra of these stimuli are highly similar, making them less discriminative compared to the other stimulus groups (periodic and quasi-periodic) (column 2 in Figure 2C). As the coherence analysis quantified the synchronization of the spectral information of two variables, for the periodic stimuli (single frequency) and even the quasi-periodic stimuli (containing multiple dominant frequencies), measuring the amount of synchronization between the narrow frequency bands (Vaid et al., 2015) was relatively less likely to be impacted by noise. However, as the chaotic stimulus spectra are similar and less discriminant (column 2 in Figure 2C) compared to the other stimulus groups, coherence analysis using the M2 approach is not recommended for chaotic stimuli. We suggest the coherence method with the M1 approach for the analysis of chaotic stimuli.

While using the M1 templates with the coherence method led to a lower accuracy than with the CCA method, there is some benefit to using the coherence method with chaotic stimuli. Specifically, unlike CCA, coherence analysis is not sensitive to the time lag between variables (Guevara and Corsi-Cabrera, 1996). Therefore, using the coherence method could reduce the training time because it removes the need to obtain training data in order to extract the time lag needed for the time domain correlation.

The accuracy values of the M2 approach were found to be relatively higher compared to M1 in the CCA analysis for the quasi-periodic and chaotic stimuli (Figure 6 and Table 1). This means that the EEG response to the chaotic and quasi-periodic stimuli may contain not only the stimulus-locked components but also more complex dynamics that did not correlate with the visual stimuli while being discriminative.

### Within-Group Subjective Visual Fatigue Rate Evaluation

From Figure 7, it can be seen that the mean VAS scores of the periodic stimulus group (P_1_ – P_4_, corresponding to frequencies of 20, 25, 35, and 40 Hz) decreased as the frequency of the target stimuli increased. These results confirm the fact that higher frequencies cause a less subjective visual fatigue level compared to lower ones (Allison et al., 2010; Volosyak et al., 2011; Yoshimoto et al., 2017). The statistical results show significant differences between all the pairs, except for P_1_ – P_2_ and P_3_ – P_4_ which were close to each other compared to the other pairs.

Q_4_ stimulus had higher VAS scores compared to Q_1_. This may be due to the fact that Q_4_ stimulus had dominant components in lower frequencies (column 2 in Figure 2B) compared to Q_1_, possibly leading to a more subjective visual fatigue. Ch_1_ stimuli caused lower subjective visual fatigue compared to Ch_3_ and Ch_4_. This could be due to the differences in the spectrum of Ch_1_ compared to those of Ch_3_ and Ch_4_ (column 2 in Figure 2C) which tend to be in the higher frequencies.

In summary, the periodic stimulus group was less favorable considering the higher subjective visual fatigue level compared to the quasi-periodic and chaotic stimulus groups. For designing homogenous BCI, it is recommended to optimize the quasi-periodic and chaotic groups’ orthogonal stimuli by evaluating their auto- and cross-correlation functions while at the same time choosing appropriate frequency bandwidths to minimize variations in the subjective visual fatigue.

### Between-Group Subjective Fatigue Rate Evaluation

The comparison of the subjective visual fatigue rates of the periodic, quasi-periodic, and chaotic stimulus groups showed that the chaotic group caused less visual fatigue compared to the other two stimulus groups. The quasi-periodic group caused lower levels of visual fatigue compared to the periodic one (Figure 8). These results indicate the superiority of using the chaotic group for designing new comfortable and ergonomic VEP-based BCIs. Our recent study also showed that visual stimuli with chaotic characteristics lead to significantly less visual fatigue (Shirzhiyan et al., 2019).

The visual fatigue reduction seen in the chaotic and even the quasi-periodic stimuli group could be due to their dynamical and complex nature which is more compatible with the visual system compared to synthesized single frequencies. It has been shown that the periodic stimuli that exist in nature are not very pure in tone and have more than a single frequency as they contain quasi-rhythmic components and have a complex dynamical structure (Kayser et al., 2003; Butts et al., 2007; Mazzoni et al., 2011).

A simple deterministic dynamical system is also able to generate extremely unpredictable, divergent, and fractal behaviors (Boeing, 2016). These behaviors contain infinitely self-similar patterns avoiding exact repetition (periodicity). It is shown that fractal images and natural patterns are more appealing compared to the synthesized ones (Redies, 2007; Chapeau-Blondeau et al., 2009; Hagerhall et al., 2015; Kardan et al., 2015), and also the chaotic patterns with high fractal dimension and Lyapunov exponent are more aesthetically pleasing (Aks and Sprott, 1996). The application of natural visual stimuli that have sparse encoding can induce a resonant state leading to the adaptation of the visual system to natural patterns (Sekuler and Bennett, 2001; Redies, 2007) and possibly lower visual discomfort. The results of the current study show that visual fatigue scores are lower for quasi-periodic and chaotic stimuli compared to periodic stimuli having one frequency. It is speculated that this could be potentially because of the adaptation of the visual system to the presented complex dynamical stimuli.

Additionally, the chaotic, quasi-periodic, and periodic stimulus groups were closer to the 1/*f* amplitude spectrum, with the chaotic group being the nearest (dotted black line in the amplitude spectra plots of Figure 2). This pattern matches our results of the VAS scores as the chaotic group had the least visual fatigue in the same order with the other two groups. The relevance of a comparatively less visual fatigue and nearness of the chaotic stimuli to the 1/*f* amplitude spectrum is in agreement with previous studies reporting that the visual system encodes stimuli with 1/*f* amplitude spectral information (Tan and Yao, 2009; Ellemberg et al., 2012; Isherwood et al., 2017; Yoshimoto et al., 2017).

### Limitations and Plans for Future Studies

Our study has several limitations. Within-group analysis of the subjective visual fatigue rates shows significant differences in all the three groups. In practice, it is not favorable that different target stimuli have different discomfort levels. To avoid possible within-group differences, the parameters of the stimulus-generated maps for each stimulus group could be selected in order to have similar stimulus spectra while at the same time preferring a higher frequency range instead of a lower one.

In this research, we did not study the optimization process for selecting the appropriate parameters of the quasi-periodic and chaotic stimuli. For future studies, optimization of the parameters with the aim of having a sharper and greater auto-correlation function of the target stimuli and a lower cross-correlation function with other stimuli should be considered. It is possible that such optimization can lead to better accuracy results.

The calculated lag times from the cross-correlation functions in three different stimulus groups represent interesting patterns. As can be seen from Figure 9, the obtained delay for each target stimulus group differed from each other mainly in the periodic stimulus group, while this delay was almost constant among the target stimuli in the quasi-periodic and chaotic stimulus groups. The diversity in the delay lags could potentially be due to the non-linear behavior of the visual system to the presented input. We plan to investigate this effect in a future project and study the reasons leading to differential system delays for different stimulus characteristics.

In our previous study, we have shown that using binary chaotic codes *versus m*-sequences could decrease the subjective visual fatigue, and this could be used as a modulating code in c-VEP-based BCIs. Additionally, in this study, we found that the chaotic stimulus group provided very high discrimination between its individual stimuli, Ch_1_–Ch_4_, and could reduce the fatigue rate better when compared to the traditional stimuli for VEP generation (periodic stimuli). For further studies, it would be probably feasible to attempt using chaotic stimuli generated from other chaotic maps (such as Hanon map) and as short as codes commonly used in c-VEP studies. This could lead to the design of more comfortable and ergonomic c-VEP-based BCIs.

## Conclusion

In this study, we introduced for the first time quasi-periodic and chaotic visual stimuli for evoking VEPs in order to use them in VEP-based BCIs and compared them with traditional periodic stimuli used in SSVEP-based studies. The presented complex stimuli (quasi-periodic and chaotic stimuli) satisfy the necessities for use as visual stimuli in VEP-based BCIs. The best target identification accuracy was obtained for the chaotic stimuli. Potentially due to the more natural-like characteristics of the chaotic and quasi-periodic stimuli, they led to less subjective visual fatigue compared to the periodic stimulus group.

## Data Availability Statement

All datasets generated for this study are included in the article/Supplementary Material.

## Ethics Statement

The studies involving human participants were reviewed and approved by the office of research review board and the research ethics committee of the Tehran University of Medical Sciences with the LREC protocol number IR.TUMS.REC.1394.2110. The patients/participants provided their written informed consent to participate in this study.

## Author Contributions

ZS: data acquisition, analysis and interpretation, and first draft of manuscript. AK: data acquisition and analysis and contribution to manuscript writing. MF: hardware design and data acquisition. ES and MG: data acquisition and analysis. AM: study design, hardware, data interpretation, and supervision. MH: data interpretation, design, overall supervision, and manuscript review. AJ: study design, funding, project supervision, and principal investigator. All authors contributed to the article and approved the submitted version.

## Conflict of Interest

The authors declare that the research was conducted in the absence of any commercial or financial relationships that could be construed as a potential conflict of interest.
