# Supplementary material for: Toward New Modalities in VEP-Based BCI Applications Using Dynamical Stimuli: Introducing Quasi-Periodic and Chaotic VEP-Based BCI
Source: Front Neurosci. 2020 Nov 17;14:534619. doi: 10.3389/fnins.2020.534619 (PMC7718037; doi:10.3389/fnins.2020.534619)
Supplement: Supplementary file 1 [file Table_1.DOCX]

**S1 Table: The p-value and Z of Wilcoxon Signed-Rank test** **with Bonferroni correction for within group comparison of VAS scores. Significant values are in red color font.**

| **Periodic group pairs** | **p value** | **Z** | **Quasi-periodic group pairs** | **p value** | **Z** | **Chaotic group pairs** | **p value** | **Z** |
| --- | --- | --- | --- | --- | --- | --- | --- | --- |
| **P_1_-P_2_** | 0.046 | 1.995 | **Q_1_-Q_2_** | 0.371 | 0.894 | **Ch_1_-Ch_2_** | 0.049 | 1.968 |
| **P_1_-P_3_** | 0.0001 | 3.644 | **Q_1_-Q_3_** | 0.839 | 0.203 | **Ch_1_-Ch_3_** | 0.0001 | 3.535 |
| **P_1_-P_4_** | 0.0001 | 4.084 | **Q_1_-Q_4_** | 0.004 | 2.85 | **Ch_1_-Ch_4_** | 0.005 | 2.795 |
| **P_2_-P_3_** | 0.0001 | 4.084 | **Q_2_-Q_3_** | 0.632 | 0.480 | **Ch_2_-Ch_3_** | 0.009 | 2.598 |
| **P_2_-P_4_** | 0.0001 | 4.477 | **Q_2_-Q_4_** | 0.008 | 2.648 | **Ch_2_-Ch_4_** | 0.022 | 2.284 |
| **P_3_-P_4_** | 0.006 | 2.726 | **Q_3_-Q_4_** | 0.011 | 2.549 | **Ch_3_-Ch_4_** | 0.57 | 0.569 |
