# Supplementary figures and images for: Toward New Modalities in VEP-Based BCI Applications Using Dynamical Stimuli: Introducing Quasi-Periodic and Chaotic VEP-Based BCI
Source: Front Neurosci. 2020 Nov 17;14:534619. doi: 10.3389/fnins.2020.534619 (PMC7718037; doi:10.3389/fnins.2020.534619)

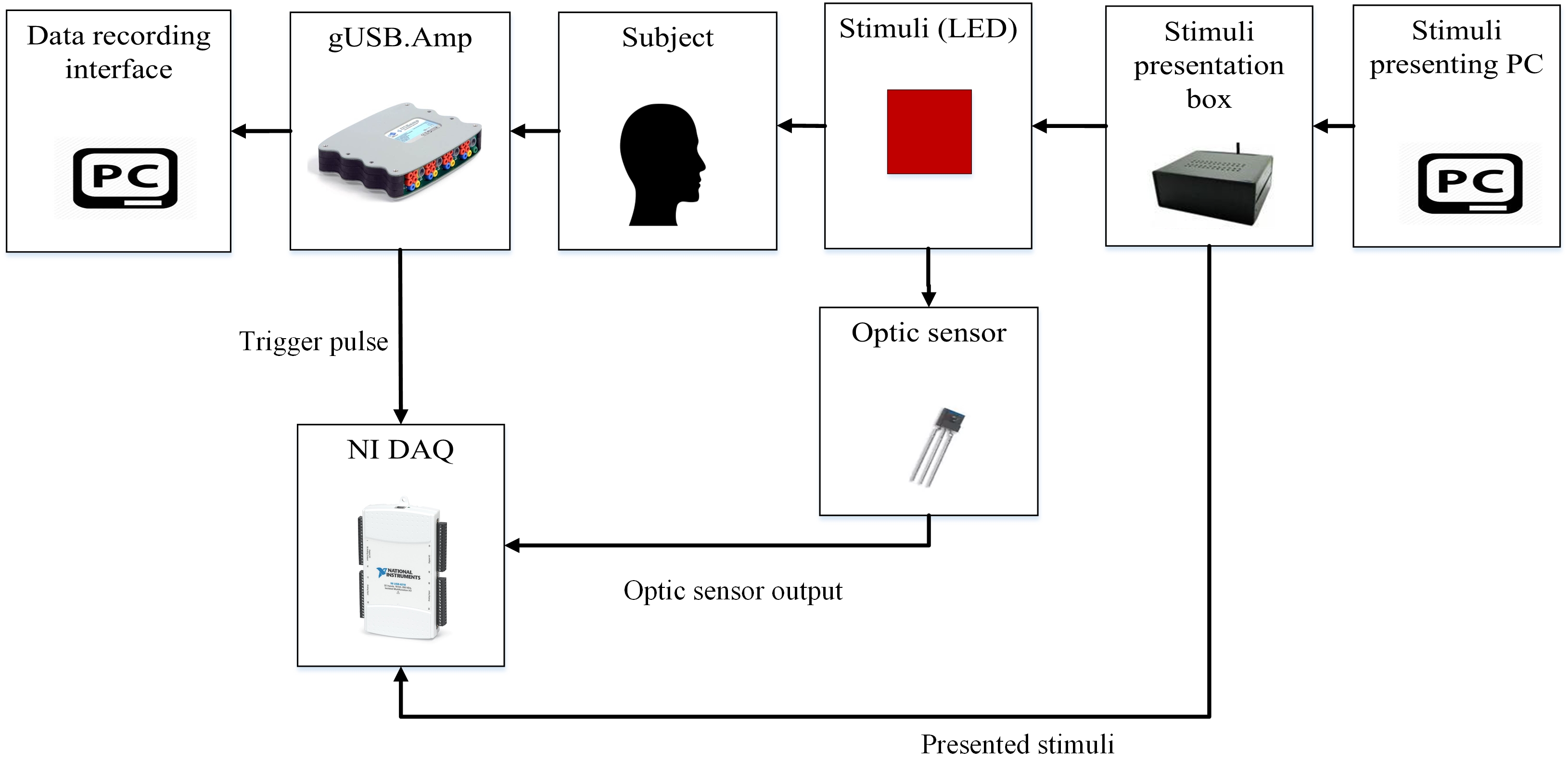

Supplement: Supplementary file 2 [file Image_1.JPEG]
